# Use of herbal medicine during pregnancy among women with access to public healthcare in Nairobi, Kenya: a cross-sectional survey

**DOI:** 10.1186/1472-6882-14-432

**Published:** 2014-11-04

**Authors:** Mamothena Carol Mothupi

**Affiliations:** School of Public Health, Moi University, PO Box 4606, Eldoret, Kenya

**Keywords:** Herbal medicine, Pregnancy, Maternal health, Concomitant medication, Kenya

## Abstract

**Background:**

Maternal health is a public health priority in many African countries, but little is known about herbal medicine use in pregnancy. This study aimed to determine the pattern of use of herbal medicine in an urban setting, where women have relatively high access to public healthcare.

**Methods:**

This cross-sectional study included 333 women attending a childcare clinic in a district public health hospital in Nairobi, Kenya, during January and February, 2012, and who had delivered a baby within the past 9 months. Qualitative and quantitative data on herbal medicine use during their latest pregnancy were collected through an interviewer-administered questionnaire. Data was analysed descriptively and the Chi square test and Fishers’ exact test used to analyse relationships among variables.

**Results:**

About 12% of women used herbal medicine during their most recent pregnancy. The use of herbal medicine was associated with a lower level of education (*p* = 0.007) and use before the index pregnancy (*p* <0.001). Only 12.5% of users disclosed such use to healthcare professionals, and about 20% used herbal medicine concomitantly with Western medicine for the same illness/condition. Women used herbal medicine for back pain, toothache, indigestion and infectious diseases, such as respiratory tract infections and malaria. A proportion of users took herbal medicine only to boost or maintain health. There were high rates of self-prescribing, as well as sourcing from family and friends. Beliefs about safety and efficacy were consistent with patterns of use or non-use, although both users and non-users were unsure about the safety and contraindications of Western medicine during pregnancy compared with that of herbal medicine.

**Conclusion:**

Herbal medicine is used by 12% of pregnant women with access to healthcare in an urban context in Kenya, and often occurs without the knowledge of healthcare practitioners. Healthcare professionals should play a role in rational use of both herbal and Western medicine, by discussing contraindications and the potential for drug-herb interactions with patients. More studies are needed into the use of herbal medicines during pregnancy, labour and the postpartum period in different geographical areas, and into the health outcomes associated with their use.

## Background

The study of herbal medicine use related to maternal health, a public health priority in many African countries including Kenya, has been limited. The use of traditional medicine, of which herbal medicine is a significant part, is relatively high in Africa: the World Health Organization (WHO) reported a prevalence of herbal medicine use of about 80% of the population [1]. Furthermore, the global use of herbal medicine is growing [2], and the industry generates billions of dollars annually in regions such as China and Europe [1, 3].

In Africa, reliance on herbal medicines is relatively high among rural populations, and is associated with a lack of access to public healthcare [1]. Use of herbal medicine may also be associated with social and cultural influences [4], perceived efficacy [5, 6], beliefs about safety, and general ease of access [6]. However, even in the context of relatively high access to public healthcare, such as in urban areas, Africans still rely on alternative or traditional systems of care [7, 8]. The use of herbal medicine when public healthcare is available has an effect on care-seeking behaviour of patients [5], rational use of drugs [2], health outcomes [9] and outcomes of care [5]. Patients with general access to Western medicine may use herbal medicine concomitantly, and often without the knowledge of a healthcare professional [6, 9, 10].

In a study in an obstetrics and gynaecology unit in a tertiary hospital in Ghana, about 50% of patients had used herbal medicine prior to admission, and the authors recommended that healthcare professionals should determine herbal medicine use among patients. It was found that use of herbal medicine was associated with low education and skill levels [10]. In a Nigerian city, researchers reported that pregnant women used both traditional herbal medicine and pharmaceutical drugs, with the highest prevalence of concomitant use among nulliparous mothers [11]. Social demographic factors, such as geopolitical zones and educational attainment, had an effect on the views of women on the safety of herbal medicine for the foetus, and side effects of herbal medicines [12]. A study in largely rural districts of Kenya found widespread use of herbal medicine among women cared for by traditional birth attendants during pregnancy, labour and the postpartum period [5]. The use of herbal medicine was often complementary to medical care, depending on barriers to healthcare access, complications, and sociocultural beliefs. According to the literature, use of herbal medicine during pregnancy, labour or the postpartum period occurs at rates ranging from 30% to 70% in a healthcare setting in urban areas of sub-Saharan Africa [10, 12, 13].

The use of complementary and alternative medicine (CAM) for maternal health has also been studied in developed countries, such as the United States [14, 15] and Norway [16]. In these contexts, the prevalence varied from 9% to 36% for herbal and other complementary therapies. In the United States, the use of herbal medicine was prevalent among rural dwellers for medicinal and nutritional purposes [15] and among those with use prior to pregnancy [14]. In developed regions, women also use other complementary and alternative therapies such as yoga, reflexology and massage for stress reduction and midwives are increasingly incorporating CAM care into their practice [17]. Use of herbal, as well as other alternative and complementary medicines in developed contexts is generally associated with higher education and cultural minorities [2]. These studies on patterns of use complement others on the clinical, regulatory and supply-oriented issues regarding traditional, alternative and complementary medicine use [2]. In both developing and developed countries, study of the use of herbal medicine (and other traditional, complementary and alternative medicines) broadens the scope of the public health perspective on not only factors that influence maternal health outcomes, but also the health system and possible areas for intervention [18].

This study was a cross-sectional survey to determine the prevalence and pattern of use of herbal medicine in pregnancy, using the World Health Organization (WHO) definition of herbal medicine. According to the WHO, herbal medicine is described as *“…herbs, herbal materials, herbal preparations and finished herbal products, that contain as active ingredients parts of plants, or other plant materials, or combinations”* [19]. Additionally, herbal medicines can be in the form of liquids, powder, capsules, tablets or ointments. Some are pre-packaged while others are prepared when needed. Herbal medicines are used not only to cure illness but to maintain or boost one’s health [19].

The study aimed to determine sociodemographic characteristics associated with herbal medicine use, aspects of health-seeking behaviour, and attitudes and beliefs about the safety and efficacy of both Western and herbal medicines among pregnant women in Nairobi, Kenya.

## Methods

Women attending a childcare clinic at Mbagathi District Hospital in Nairobi, with infants no more than 9 months old, were invited by healthcare professionals to take part in the study. Mbagathi District Hospital was the only fully operational public healthcare facility in the Nairobi area mandated to provide an Integrated Management of Childcare and Illness clinic at the time of study (according to the Ministry of Health online registry of health facilities in Kenya). All the respondents were briefed on the definition of herbal medicine by the research assistants and gave signed consent to participate in the study. Only participants above 18 years of age and the biological mothers of infants participated in the study. After informed consent was obtained, data were collected using a semi-structured, interviewer-administered questionnaire to gather qualitative and quantitative information about sociodemographic characteristics, patterns of medicine use, and beliefs about safety and efficacy of medicines. The definition of herbal medicine was included in the questionnaire and given to participants at the beginning of the interview.

The WHO estimated that 80% of African patients used herbal medicine, and thus respondents were needed for statistical analysis of data [1]. A more conservative sample size was used because studies have suggested a lower prevalence of herbal medicine in urban areas in Africa, though there is little data on herbal medicine use among urban women. A previous study suggested 40% use among obstetric patients in an urban African context [7]. At a 50% prevalence rate for the use of herbal medicine, the conservative sample size was estimated at 384 respondents. The formula used for determining sample size for this study was n = z^2^pq/d^2^, where n = number of respondents, z = value of the test statistic, p = the estimated proportion of use of herbal medicine, q = 1- p and d = degree of accuracy (5%). Of the targeted women, 337 (87%) agreed to take part in the survey. An analysis of non- response could not be performed because data for the non-respondents was not available. However, there was a high rate of inclusion.

After excluding incomplete questionnaires, data of 333 women (86% of those targeted) were included in the analysis. Data were collected over 2 months during January and February 2012.

Quantitative data are presented as descriptive statistics and were analysed by the Chi-square test at ∝ = 0.05 significance level using SPSS v16 (SPSS Inc., Chicago, IL, USA). Qualitative data were analysed using Microsoft Excel 2010. All the respondents were briefed on the definition of herbal medicine by the research assistants and gave signed consent to participate in the study. Only participants above 18 years of age, biological mothers of infants and who gave full informed consent participated in the study. The definition of herbal medicine was included in the questionnaire and given to participants at the beginning of the interview.

Authorization to conduct the study was obtained from Mbagathi District Hospital. The proposal was approved by the Ethics Committee of Moi University Institutional Review Board.

## Results

### Prevalence of use and sociodemographic characteristics of respondents

The majority of the 333 respondents were younger than 30 years of age (58.0%), were educated to secondary level or higher (68.5%), were married (67.3%), and had attended antenatal care three times or more (96.7%). There was variability in terms of number of children and distance to a health facility. Forty respondents (12.0%) had used herbal medicine during the index pregnancy (less than 9 months ago), while the number of respondents who had ever used herbal medicine was 138 (41.4%). There was an association between use of herbal medicine prior to the pregnancy and use during the pregnancy (*p* <0.001); among those who used herbal medicine before the index pregnancy, 26.8% also used it during pregnancy, compared with 1.5% of those who had never used herbal medicine. The proportion of respondents who used herbal medicine during pregnancy also significantly decreased with increasing level of formal education (*p* = 0.007) (Table 1).

**Table 1:** **Association of sociodemographic characteristics with herbal medicine use during pregnancy**

	Use of herbal Medicine during pregnancy (N =333)		Chi-square analysis	
	*Yes n (%)*	*No n (%)*	*X* ^*2*^ *, df*	*p-value*
**Age**				
< 30 y	21 (10.9)	172 (89.1)	0.556, 1	0.497
≥ 30 y	19 (13.6)	121 (86.4)		
**Education**				
no formal education	4 (50)	4 (50)	12.081, 3	0.007
primary education	13 (13.4)	84 (86.6)		
secondary education	17 (10.7)	142 (89.3)		
tertiary education	6 (8.7)	63 (91.3)		
**Number of children**				
one child	10 (9.8)	92 (90.2)	2.547, 2	0.288
two children	13 (10.3)	113 (89.7)		
three or more children	17 (16.2)	88 (83.8)		
**Marital status**				
never married	7 (8.3)	77 (91.6)	4.475, 2	0.107
married	27 (12.1)	197 (87.9)		
not currently married	6 (24)	19 (76)		
**Distance to health facility**				
not far, <5 km	11 (10.7)	92 (89.3)	0.998, 2	0.614
somewhat far, 5–10 km	13 (10.8)	107 (89.2)		
far, >10 km	16 (14.5)	94 (85.5)		
**Antenatal care attendance**				
twice or less	3 (30)	7 (70)		Fishers’ Exact: 0.100
three times or more	35 (11.2)	287 (88.8)		
**Ever use before index pregnancy**				
yes	37 (26.8)	101 (73.2)	48.837, 1	< 0.001
no	3 (1.5)	192 (98.5)		

### Indications for use

**Figure 1:**
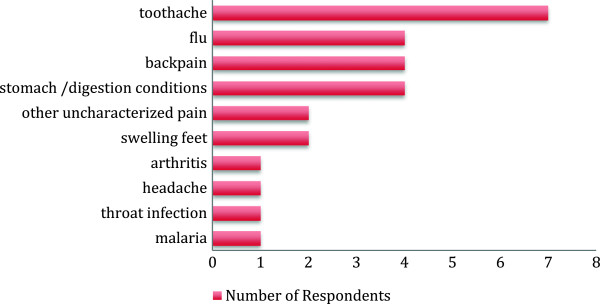
**Indications for herbal medicine use during pregnancy.**

The indications for use of herbal medicine during pregnancy included toothache, various types of pain, flu and stomach problems (Figure 1). The conditions indicated included common ailments such as malaria and respiratory tract infections, as well as pregnancy-related conditions such as swollen feet, back pain, and digestive problems. Prescribed pharmaceuticals were concomitantly used by 22% of users to boost health and 29% to cure illness.

### Health-seeking behaviour

Although some respondents used prescribed pharmaceuticals and herbal medicine concomitantly, only five (12.5%) had revealed such use to a healthcare professional. When use of herbal medicine was disclosed, four respondents reported that the healthcare provider advised them about side effects (n = 1), discouraged such use (n = 1) or showed indifference (n = 2).

**Figure 2:**
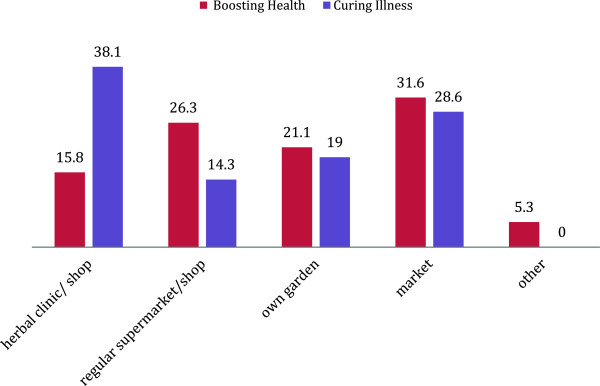
**Source of herbal medicine based on general indication of use (% users).**

**Figure 3:**
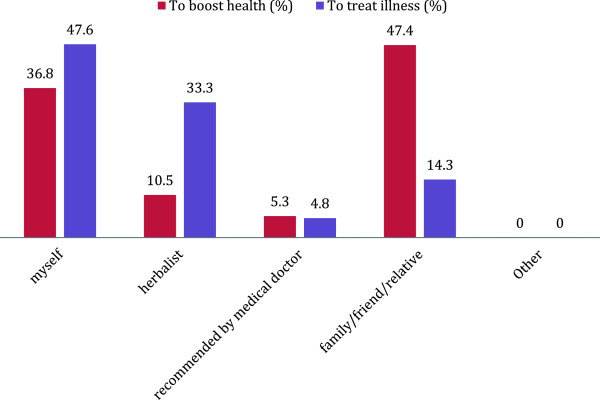
**Prescription source of herbal medicine during pregnancy.**

Herbal medicine was largely acquired from markets (32%) for health boosting purposes, and herbal clinics (38%) to treat illness (Figure 2). Herbal medicine was largely acquired on the advice of family or friends when the respondent was not ill to boost health (47%), while herbalists were largely consulted to treat illness (33%) (Figure 3). There were high rates of self-prescribing among respondents in the use of herbal medicine to boost health (37%) and to treat illness (47%).

**Figure 4:**
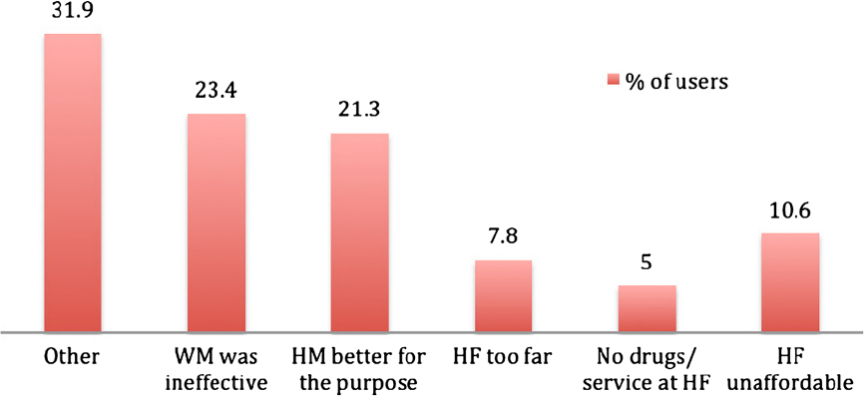
**Reasons for** ***ever use*** **of herbal medicine.** WM: Western medicine; HM: Herbal medicine; HF: Health facility.

Among reasons for *ever use* were “other” (32%), a perception that Western medicine was “not working” (23.4%), and that herbal medicine was better or more effective for that illness/condition (21.3%). Less common reasons were affordability (10.4%), distance to a healthcare facility (7.8%), and lack of drugs (5.0%) or service (1%) at a healthcare facility (Figure 4). “Other” reasons for herbal medicine use included preference (28.8%), experimentation (17.7%) with herbal medicine and advice of family or friends (11.1%).

### Perception about safety and efficacy

There were differences between users and non-users in terms of perception of safety and efficacy of herbal medicine during pregnancy (Table 2). Perception of safety of herbal medicine during pregnancy was consistent with use/non-use. For instance, the majority of users of herbal medicine tended to agree and non-users to disagree that herbal medicine was safe during pregnancy (*p* <0.001). The perception of safety altered slightly when a medical doctor hypothetically recommended herbal medicine. Users agreed (92.3%) that herbal medicine could have health benefits if recommended by a medical doctor, while non-users also agreed (51.8%) or were unsure (26.2%, *p* <0.001).

**Table 2:** **Perception of safety and efficacy of herbal medicine and Western medicine (particularly pharmaceuticals) during pregnancy**

	Agree (%)		Disagree (%)		Not sure (%)		Pearson-Chi Square		
	User	Non-users	User	Non-user	User	Non-user			
**Safety of herbal medicine**							*χ* ^2^	*df*	Exact *p*-value
Most Western medicine not safe for me (mother) during pregnancy	15	10.6	85	85.7	0	3.8	2.122	2	0.358
Most Western medicine not safe for my baby during pregnancy	15.0	10.2	85	86	0	3.8	2.244	2	0.314
Most herbal medicine is not safe for me during pregnancy	27.5	65.2	42.5	5.5	30	29.3	56.315	2	< 0.001
Most herbal medicine is not safe for my baby during pregnancy	27.5	64.5	42.5	4.8	30	29.7	54.449	2	< 0.001
Most herbal medicine is natural	77.5	26.3	12.5	45.7	10	28	42.191	2	< 0.001
Most herbal medicine is safe	71.8	20.8	10.3	45.7	17.9	33.4	46.400	2	< 0.001
**Efficacy of herbal medicine**									
Herbals beneficial if recommended by doctor	92.3	51.8	2.5	21.8	5.1	26.2	22.491	2	< 0.001
Herbals beneficial if recommended by herbalist	46.1	14.6	38.4	57.3	15.3	27.9	22.849	2	< 0.001
Herbals beneficial if recommended by family/relative	69.2	17	12.8	55.3	17.9	27.6	53.947	2	< 0.001
There are illnesses or conditions for which herbal medicine is more effective than Western medicine	89.7	27.3	5.1	49.1	5.1	23.5	59.367	2	< 0.001
There are illnesses or conditions for which Western medicine is more effective than herbal medicine	97.4	87	2.6	2	0	10.9	4.725	2	0.079

Users tended to believe in the efficacy of herbal medicine over Western medicine/prescribed pharmaceuticals for some illnesses and conditions, while non-users disagreed with the statement (*p* <0.001). In contrast, both users (97%) and non-users (87%) agreed with the statement that Western medicine is more efficacious than herbal medicine for some illnesses or conditions (*p* = 0.079).

## Discussion

Respondents in this study had high rates of antenatal care attendance, which indicates relatively high use of public healthcare during pregnancy, and is comparable with statistics from other facilities in Nairobi [20]. The high use of public health facilities presents an opportunity to discuss the use of herbal medicine with women while attending antenatal care or even during delivery. About 12% of these respondents used herbal medicine during pregnancy, and this estimate is lower than others in sub-Saharan Africa. There is an information bias associated with lack of disclosure of herbal medicine use to health professionals and researchers, particularly when a respondent is interviewed in a healthcare setting [13]. The rate of ever use (40.5%) among respondents was as high as that seen in another study in an urban area of Kenya (50%) [7].

As the findings indicate, herbal medicine use during pregnancy should be considered not only in terms of pregnancy-related conditions, but also other common illnesses (Figure 1). There were various reasons for ever use of herbal medicine, related to access to health services and the sociocultural environment of the respondents. Health service-related reasons included the cost, inaccessibility of health facilities at the time of illness, the distance to be travelled to access care, or lack of medication availability (Figure 4). Focussed discussions with women who used herbal medicine in pregnancy and during labour and the postpartum period indicated that herbal medicine was generally considered cheaper, although some users found that herbal medicine could be costlier than Western medicine (data not shown). These data suggested that cost may not be a major influence for herbal medicine use, and indeed seeking herbal or Western medicine appeared to depend on the type of illness and severity of illness.

The majority of respondents cited “Other” reasons for use of herbal medicine, as well as issues related to perception of safety and efficacy. Family and friends represent the social and cultural environment in which the pregnant woman lives and in part influence health-seeking behaviour during pregnancy. This study found that family and friends were an important source of herbal medicine (Figure 3) and users tended to trust the benefits of use if recommended by close acquaintances (Table 2).

Educational status was significantly associated with use of herbal medicine during pregnancy (*p* = 0.007). Women with no formal or only primary education used herbal medicine more than women with secondary or higher education. The higher educational attainment in this study group may also explain the lower prevalence of herbal medicine use than in other studies and among the general population of women in Nairobi. Findings of other studies indicated that educational status is a consistently important factor in the use of herbal medicine and other alternative systems of care [2, 10, 13].

About 20% of herbal medicine users during pregnancy also sought pharmaceutical medicine for the same illness or condition, and the majority did not disclose herbal medicine use to a healthcare professional. There were also high rates of self-prescribing of herbal medicine among users (Figure 4), suggesting that users like to take control of their own health [6, 9]. The perceptions of safety and efficacy were consistent with use or non-use of herbal medicine. Many pharmaceutical drugs and Western medicine interventions have safety information and guidelines for use during pregnancy; essentially, both herbal and pharmaceutical medication should be contraindicated during pregnancy and their safety and efficacy determined [13, 21, 22]. All respondents (users and non-users) should be equally wary of taking herbal or Western medicine during pregnancy and consult a reliable source of information on their safety and efficacy. Perception of safety and efficacy may also be influenced by prescription source. Medical doctors, family and friends and herbalists have different knowledge and experience of herbal medicine and this may influence the perceived health benefit that can be acquired from the herbal medicine they recommend.

There are limitations to this study. This is a descriptive study of the use of herbal medicine among women with good access to public healthcare in Nairobi. Thus, the sample may not be fully representative of the pregnant female population in Nairobi, nor those who access different types of healthcare (e.g., private and informal).

## Conclusion

The health-seeking behaviour of women who use herbal medicine during pregnancy suggests that they rely on it as a resource even if public health facilities are available. Thus, there is an opportunity for the involvement of healthcare providers in patient education about the appropriate use of herbal medicine and pharmaceuticals. There is inadequate knowledge among respondents about the safety of both herbal and Western medicine during pregnancy. This study indicates that there is a necessity for women to be adequately informed of the potential risks of concomitant use of herbal medicine and pharmaceutical drugs during pregnancy.

This study recommends further research in the use and patterns of use of herbal medicine among women during pregnancy, labour and the postpartum period, in both public and private health facilities, and should be extended to rural areas. Further studies on specific herbal and traditional medicines, and longitudinal studies examining health outcomes for mothers and babies after use of herbal and other alternative therapies, are warranted.

## Abbreviations

CAM: Complementary and Alternative Medicine
WHO: World Health Organization.
